# Evaluation of the prophylaxis and treatment of COVID-associated coagulopathy

**DOI:** 10.1186/s40545-020-00274-8

**Published:** 2020-10-26

**Authors:** Ali Elbeddini, Rachel Gerochi, Ahmed Elshahawi

**Affiliations:** 1 Winchester District Memorial Hospital, 566 Louise Street, Winchester, ON KK0C2K0 Canada; 2 Leslie Dan Faculty of Pharmacy, University of Toronto, 144 College st, Toronto, M5S 3M2 Canada; 3 University of Toronto Medical School, U 1 King’s College Circle, Medical Sciences Building, Room 2109, Toronto, ON M5S 1A8 Canada

## Abstract

Most of the current “literature” surrounding the presence of thrombosis in COVID-19 disease and appropriate prophylaxis/treatment modalities is certainly retrospective at best, and anecdotal at worst. But in these times of rapidly changing information and perspective, an assessment of all available data (including expert opinion) is the goal of this review. Bleeding risk factors for COVID-19-associated bleeding may include other systemic diseases, including hypertension, diabetes, cardiovascular disease, and immunosuppression. Individuals with hypertension should not discontinue their medication. Current evidence does not support changes in the management of hypertension. As COVID-19 progresses, coagulation pathways are activated as part of the host inflammatory response to limit the viral infection. Specifically, D-dimers, products of fibrin as it is degraded within clots, are elevated in many cases of hospitalized COVID-19 patients. D-dimers are an indicator of a clot (thrombus) formation and breakdown. More severe COVID-19 disease may lead to overt disseminated intravascular coagulation (DIC), associated with high mortality. DIC is a coagulopathy that may arise from the systemic inflammatory response to the virus and damaged tissue caused by the infection. Bleeding risk factors may include other systemic diseases, including hypertension, diabetes, cardiovascular disease, and immunosuppression. Individuals with hypertension should not discontinue their medication. Current evidence does not support changes in the management of hypertension. As COVID-19 progresses, coagulation pathways are activated as part of the host inflammatory response to limit the viral infection. Specifically, D-dimers, products of fibrin as it is degraded within clots, are elevated in many cases of hospitalized COVID-19 patients. D-dimers are an indicator of a clot (thrombus) formation and breakdown. More severe COVID-19 disease may lead to overt disseminated intravascular coagulation (DIC), associated with high mortality. DIC is a coagulopathy that may arise from the systemic inflammatory response to the virus and damaged tissue caused by the infection. My manuscript presents the risk and evidence around the COVID-19-associated coagulopathies

## Background

One of the most common clinical findings in COVID-19 patients is the occurrence of thrombotic events despite thromboprophylaxis, leading to poorer outcomes and more ICU transfers. Although uncertain, there is a large consensus that hypercoagulability accompanying COVID-19 infection is the result of inflammatory responses to the virus that lead to coagulation pathways stimulation. Current literature concerning this coagulopathy and its prophylaxis/treatment in the COVID-19 population is certainly retrospective at best, and anecdotal at worst. In these times of rapidly changing information and perspective, an assessment of all available data, including expert opinion, is the goal of this commentary.

## COVID-19-associated coagulopathy

**Fig. 1:**
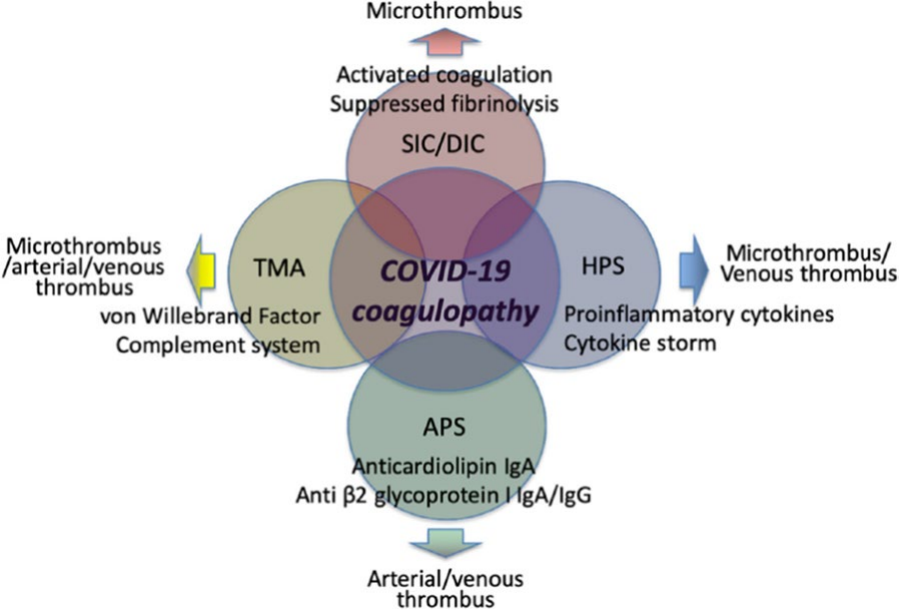
Characteristic features of COVID-19-associated coagulopathy

Coagulopathy associated with COVID-19 has been described as similar to other pro-inflammatory disorders including anti-phospholipid syndrome, however, can be characterized as its own classification (Fig. 1) [1]. As COVID-19 progresses, coagulation pathways are activated during the host inflammatory response to limit the viral infection and create a procoagulant effect. Coagulopathy does not appear to stem from thrombotic properties of the virus itself, although this currently remains debateable [2–4]. This inflammatory response together with damaged tissue caused by the infection, in more severe COVID-19 cases, may lead to overt disseminated intravascular coagulation (DIC) and consequently an increased risk of mortality. Early reports indicate a DIC-like picture, although most describe issues strictly with clotting rather than having a bleed component [2, 5, 6]. Recent evidence has additionally shown a risk to both large vessels and potential wide-spread micro-thrombosis in several organ systems which may be a concern for end-organ damage and oxygenation. The latter may prove difficult in terms of diagnosing, and the former has challenges due to the risk of additional staff exposure for testing [7].

Incidence of thrombotic events have been as high as 25–50% in COVID-19 patients admitted to the ICU [8–10], with an increasing severity of illness correlated to a higher occurrence of these events. In comparison to deep vein thrombosis (DVT), pulmonary embolism (PE) is the more common of the two in this patient population [11]. Observations of terminal digits in some COVID-19 patients have shown vasculitis, which can potentially lead to this increase in PE and consequently worsened hypoxemia [12]. Most literature on the increased VTE incidence and thrombosis in the COVID-19 population come from Chinese literature where standard pharmacological thromboprophylaxis is not routinely provided [13], so there may be a bias of increased VTE coming from initial reports. It is important to note that more recent studies still observe relatively high rates of thrombotic events despite appropriate thromboprophylaxis [11]. Alternatively, the increase could be just a simple issue of earlier experience, observation, and time to study compared to the US and Europe. A multicentre study from the US studying 400 COVID-19 patients found the rate of radiographically confirmed VTE to be 4.8% and 7.6% in general wards and critically ill patients, respectively. All patients were reportedly on standard doses of prophylactic anticoagulation. In comparison to studies originating in China and Europe, these rates are much lower which may be attributed to the lack of a consistent protocol for imaging patients suspected of VTE. As a result, thrombotic events may have been missed and under reported [14]. Regardless of the incidence of VTE in this population, there is no question that there is significant morbidity and mortality due to COVID-19 associated coagulopathy.

## Hypercoagulability markers

Beyond retrospective analyses, there are many case reports and anecdotal evidence from colleagues around the United States and globally indicating an observance of at least an increase in hypercoagulability markers, if not VTE itself. The debate over which labs or other findings to use as objective measures of risk assessment continues. Many institutions have developed formal protocols using D-dimer and fibrinogen levels, for example, while others have continued to assess based on patient-specific risks.

**Fig. 2:**
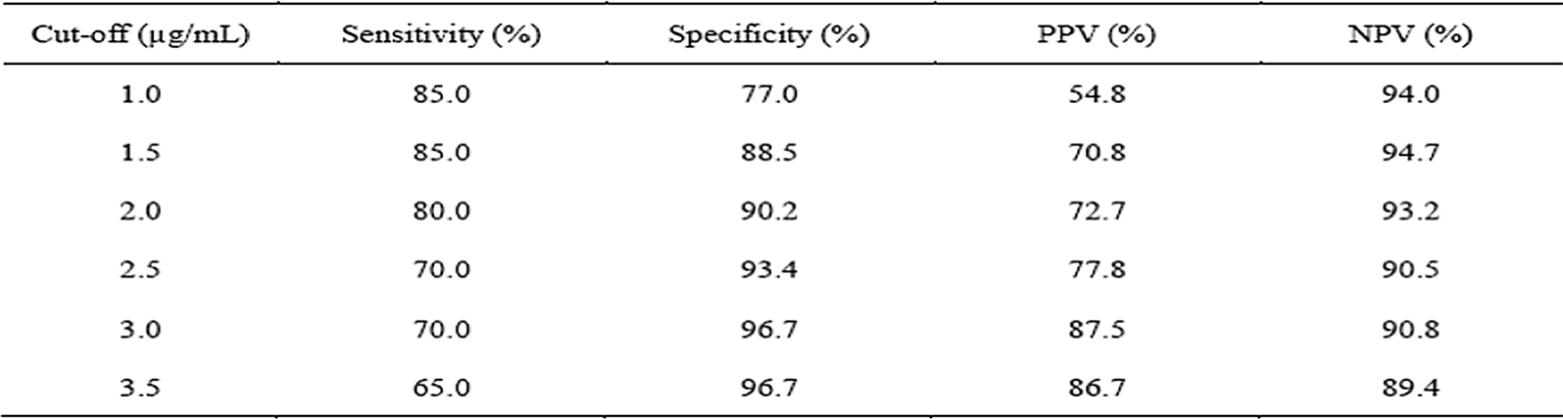
Sensitivity, specificity, positive predictive value (PPV) and negative predictive value (NPV) of different D-dimer cut-off levels for predicting VTE in COVID-19 patients [19]

In many hospitalized COVID-19 patients, particularly ones characterized as “severe”, there seems to be an increase in D-dimer levels. (We have seen up to the 20,000 range here.) An Italian study showed an average D-dimer of 4800, with all 24 patients studied exhibiting levels greater than normal [15]. Furthermore, a French report of VTE events looked at a prospective series of 150 patients with ARDS in the ICU, comparing it with a matched non-COVID historical ARDS cohort. Greater than 95% of the COVID cohort had a significantly elevated D-dimer and fibrinogen levels. Imaging was done for suspicion of thrombotic events and about 43% of COVID-19 patients had relevant VTE events. PE occurred in 25% of those with respiratory decline or rising D-dimer [16]. Elevated D-dimer (along with other elevated fibrin degradation products) was linked as a predictor of mortality in one single-center retrospective Chinese study; the average D-dimer for non-survivors was almost four times the survivor group [15]. A second dual-hospital retrospective study of 250 patients confirmed this, showing mortality linked with a D-dimer over 1000 [17]. A report of three cases of clinically significant coagulopathy resulting in multiple infarcts in a Chinese hospital detailed all three patients as having a D-dimer > 2000 (one with 21,000). These patients also all had elevations in anti-phospholipid antibodies [18]. In another single-center retrospective study, D-dimer had a PPV for the development of VTE of > 70% once the level reached 1500 ng/ml (Fig. 2) [19]. High levels of D-dimer level may also be a useful marker of pulmonary fibrin deposition, an observation commonly documented in several other lung diseases such as ARDS [11]. In a single-site evaluation, a cohort with ARDS (about 42%) had D-dimer and PT elevations to a statistically significant degree compared to the non-ARDS group. However, neither were elevated to what most of us would consider a clinically significant degree [20].

There is also evidence of increases in fibrinogen and CRP in COVID-19 patients according to many reviews [21]. Since we typically see severe disease in the ICU, it is difficult to tease out from these observed elevations inflammatory effects versus clinically significant effects on the coagulation cascade [13, 16]. Besides this, there is a concomitant increase in von Willebrand Factor, Lupus anticoagulant, and Factor VIII [16]. These elevations, however, are still associated with critical illness, thrombosis and mortality [14]. Due to the lack of specificity, it is difficult to use this as a measure of thrombotic risk outside of trending for general inflammatory assessment.

Interestingly, platelets tend to be WNL on admission in the majority of COVID-19 patients, although they may trend significantly lower in severe disease [22, 23]. Some researchers have suggested clinicians monitor PTT, platelet count and D-dimer concentrations every 2–3 days [22]. In one report, PTs were slightly elevated in just over 50% of patients (although not very significantly), and PTT was not statistically elevated on admitting in with an average result of just 31. In another, both PT and PTT were WNL in 197 patients, and PT was not elevated significantly elevated in a third [15, 17, 19]. PTT and PT were elevated in one evaluation of 24 patients in Italy, but again not to an appreciable degree [15]. There have been several reports, however, of PTTs being unreliable for assessing heparin therapy in these patients; Xa levels may be more accurate. Neither PT nor PTT seems to be a reliable value in assessing general coagulopathy in this population.

One study out of Italy reviewed TEG results from 24 patients admitted to an ICU with COVID-19 and ARDS and matched them with the results of 40 healthy adult comparators. By comparison, R (50% of COVID patients) and K (83%) values were shortened significantly, while MA (83%) was increased, and LY-30 (100%) was considerably lower in the COVID patients. It indicated that all patients had a state of hypercoagulability, although the authors did not characterize it as DIC [15]. Another investigation from Italy exhibited similar results [24].

## Thromboprophylaxis and treatment

**Fig. 3:**
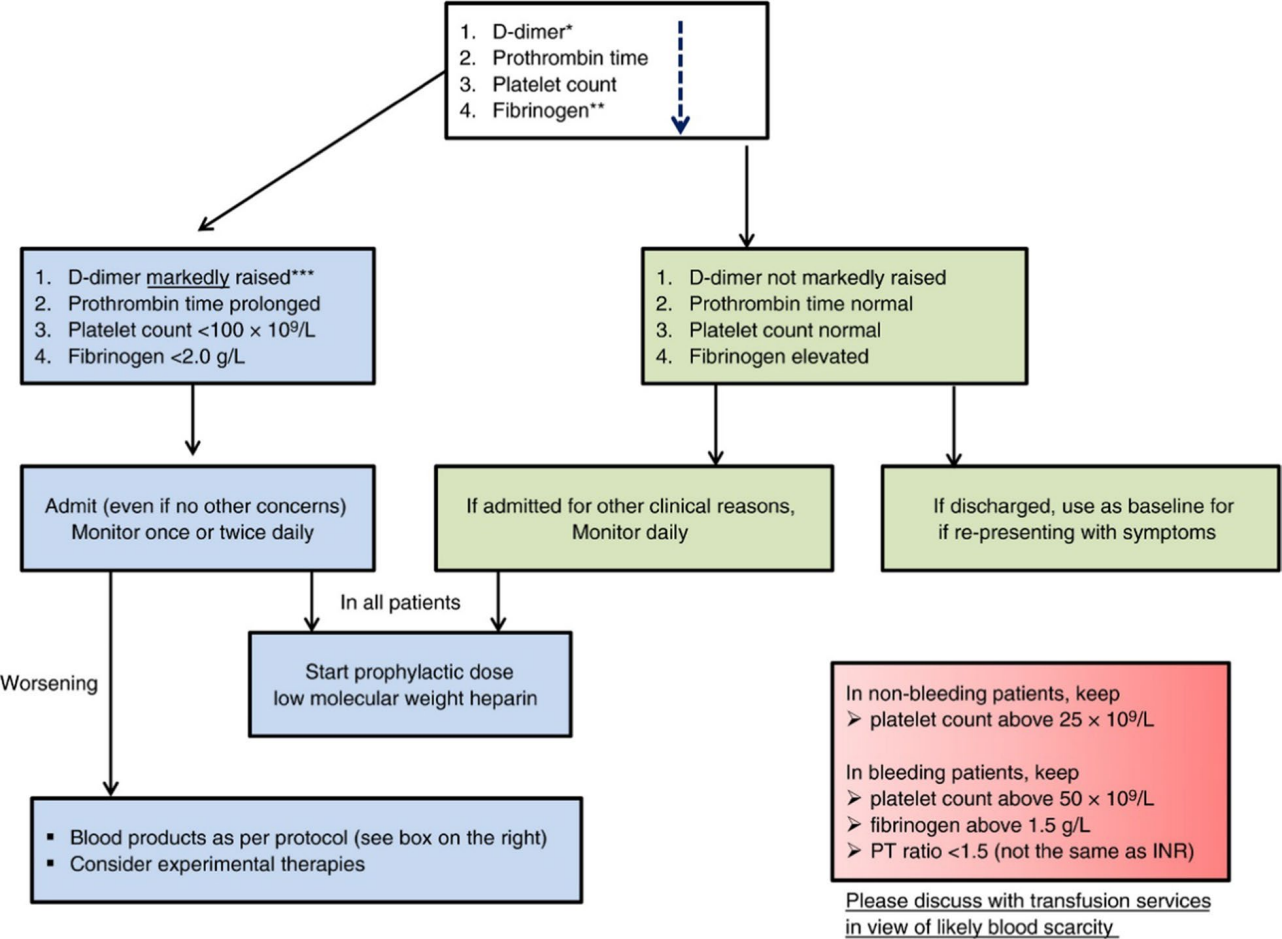
ISTH algorithm for management of coagulopathy in COVID-19 [33]. Algorithm for the management of coagulopathy in COVID‐19 based on simple laboratory markers. * The list of markers is given in decreasing order of importance. ** Performing fibrinogen assays may not be feasible in many laboratories, but monitoring the levels can be helpful after patient admission. *** Although a specific cut‐off cannot be defined, a three‐ to four‐fold increase in D‐dimer values may be considered significant. Anyone of the values in this table may be regarded as significant

There is a continued debate regarding which patient populations should get which pharmacological intervention: full dose versus standard prophylaxis or even an increased prophylactic dose. The ISTH Guidelines recommend prophylaxis with LMWH for all COVID-19 patients barring contraindication (PLT < 25, or active bleeding), although they fail to indicate the recommended dosing [25]. They also provide a flowchart for assessing admission of COVID-19 patients that essentially indicates using only prophylactic LMWH for “markedly” elevated D-dimer unless an alternative diagnosis exists (Fig. 3).

**Fig. 4:**
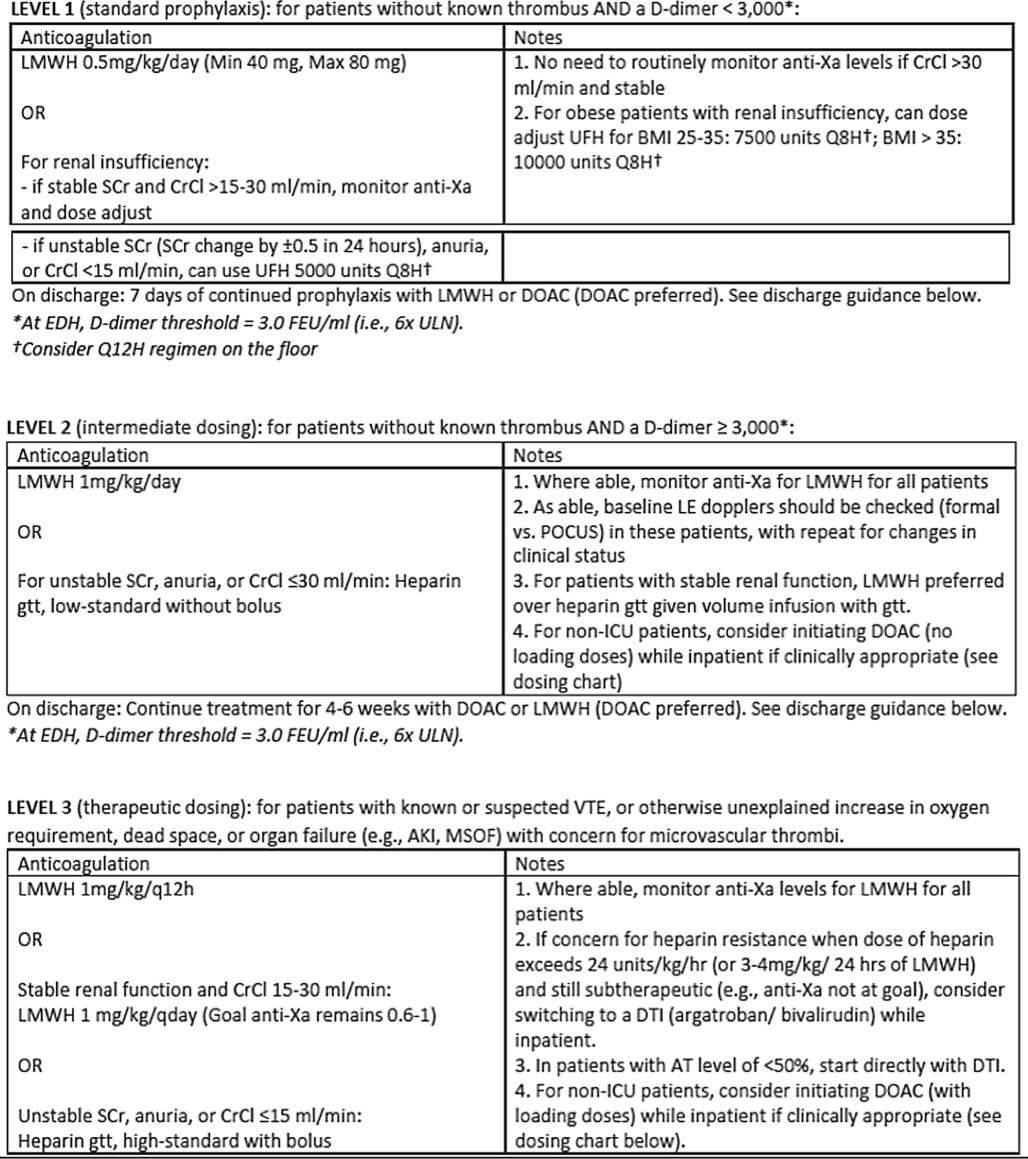
Emory protocol—April 22, 2020

**Fig. 5:**
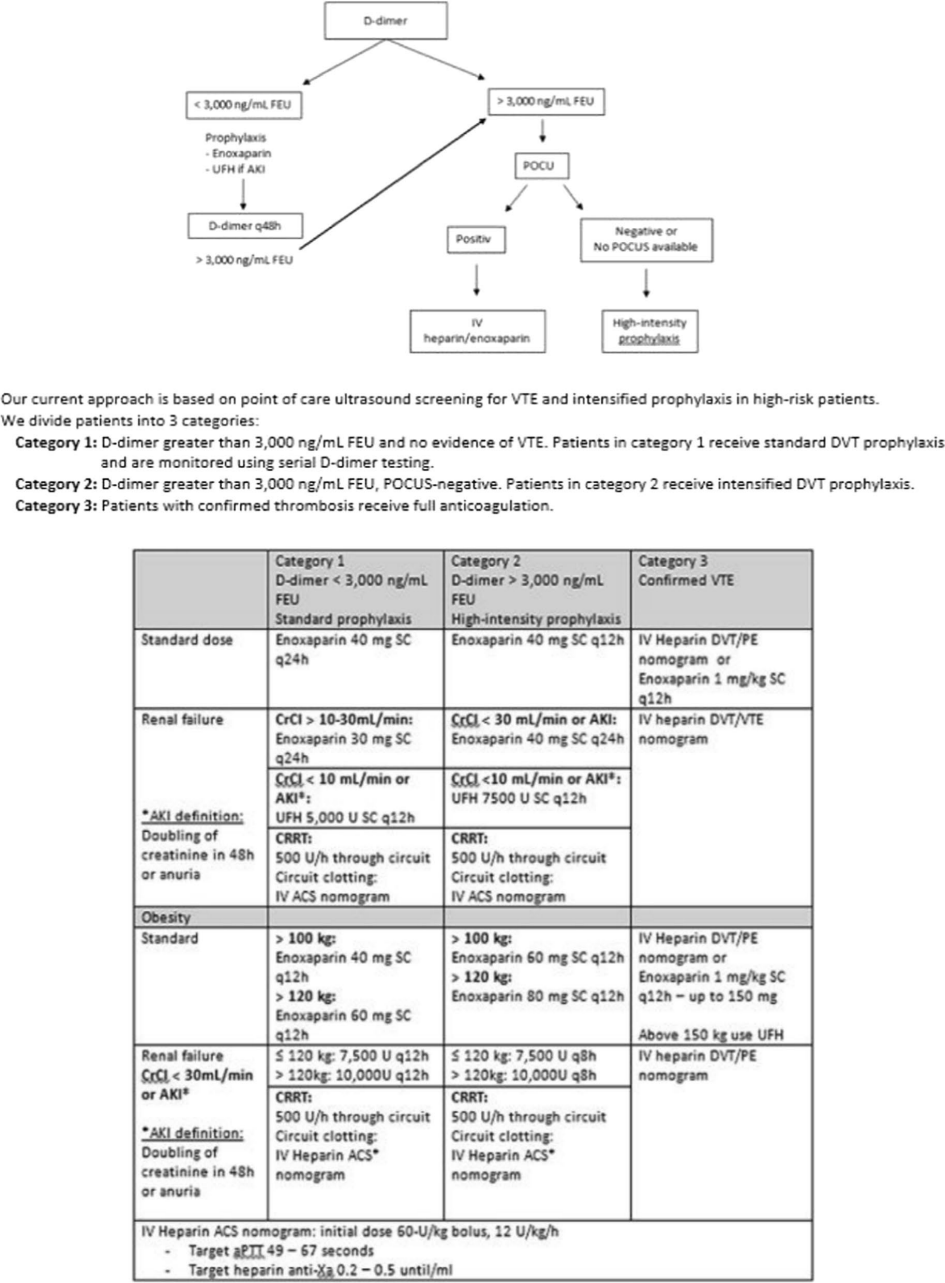
Cleveland Clinic thrombosis risk assessment [36]

The pharmacological doses required to help abate thrombosis in these patients is not well established. Studies and consensus statement/opinion papers have been inconsistent across the board, with proposed prophylactic doses of enoxaparin ranging from 40 mg/day to 0.5 mg/kg Q12H. Possibly most concerning is the fact that thrombosis is likely occurring despite anticoagulation even at therapeutic doses [26, 27]. Autopsies performed on 10 COVID-19 patients showed the presence of microthrombi in lung tissue, a possible marker of in situ pulmonary thrombosis. With the disproportionate occurrence of pulmonary clotting events without an increase in DVT, this may have implications on the appropriate therapy for COVID-19 patients and whether anticoagulation is indeed the best course of action [11]. Cleveland Clinic and Emory have taken a tiered approach to management based on D-dimer and known thrombus (Figs. 4, 5) [20, 28]. Whether to prophylactically or fully anticoagulated patients who have no overt VTE is certainly still up for debate, with only a few societies providing initial guidance on dosing [14, 25, 29]. Outside of these, only small studies or opinion papers have touched on this topic as of yet. More will hopefully come. A current open-label study is being conducted in Switzerland to assess high versus low-dose anticoagulation in COVID-19 patients and its effects on venous and arterial thrombosis, DIC, and mortality [10].

**Fig. 6:**
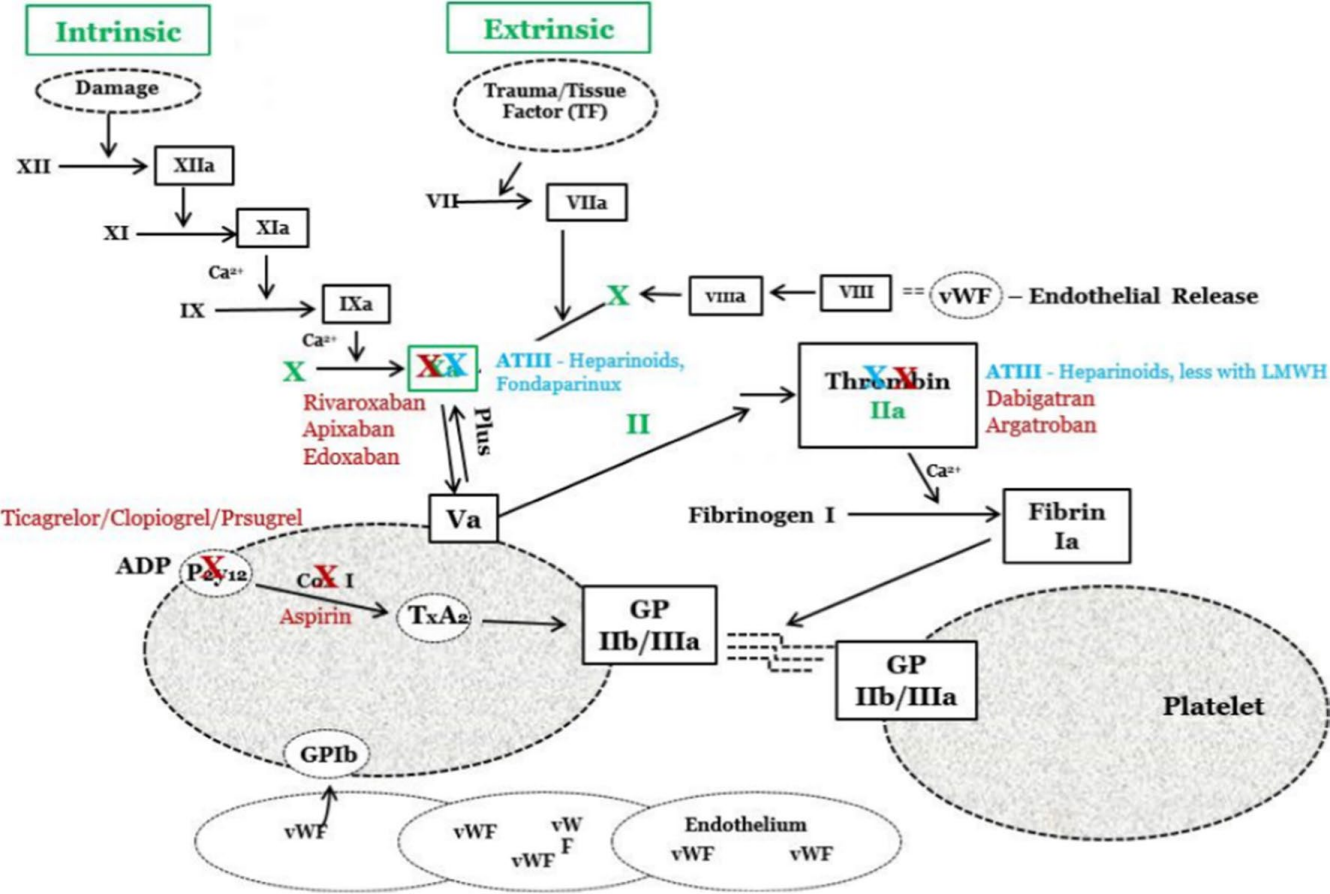
Coagulation cascade

Concerning heparin use, PTTs may be erratic, making use of them as a monitoring tool for full-dose unfractionated heparin (UFH) problematic. It may be prudent to consider using a Xa-based adjustment protocol if baseline PTTs are abnormal (pharmacists can help with this). An additional concern is that anti-thrombin three (AT-III) seems to be either down-regulated or simply reduced by consumptive processes from micro-emboli [28, 30]. Our stores of AT-III are absolutely essential for both UFH and low-molecular-weight heparin (LMWH) to bind to both thrombin and Xa, resulting in their subsequent anticoagulant effect (Fig. 6). AT-III is also used by fondaparinux (a direct Xa inhibitor). It is not required by other direct Xa inhibitors (rivaroxaban, apixaban) or direct thrombin inhibitors (argatroban, dabigatran). If AT-III is not present in sufficient amounts, the effect of heparinoids will be significantly blunted. Experimental models have shown the direct binding of heparin to the SARS-CoV spike protein 1, which is the viral anchor site for its interaction with ACE2. By binding to this protein, heparin can block the virus’ entry into the cell. These effects lack evidence in clinical practice [31].

Conversely, UFH has been shown to exhibit anti-inflammatory effects [32, 33], and there is also some evidence that LMWH may do the same [8, 34]. This could prove beneficial beyond anti-thrombosis in this patient population with well-established hyper-inflammatory states.

Regarding the use of thrombolytics: in a pre-guideline consensus statement provided by Chinese and French physicians that was just published on 4/21/20, recommendations include the use of thrombolytic rescue therapy for severe cases of COVID-19 with abrupt/severe hypotension and/or hemodynamic deterioration in combination with findings from bedside Echo [35]. The use of alteplase in non-COVID ARDS has been described in the literature previously based on the intertwined processes of inflammation and coagulation [36]. Some have recommended in COVID patients with acute decompensation using alteplase 25 mg bolus over 2 h, followed by 25 mg over the next 22 h (or about 1 mg/h for the remainder of the 50 mg dose) [37]. There have been discussions around the use of other anticoagulant modalities such as plasma exchange to treat thrombotic microangiopathy. Theoretically, the delivery of high volumes of plasma may result in the replacement of missing factors such as complement proteins and the removal of excess inflammatory mediators. More evidence is required to evaluate the efficacy of this treatment, preferably in a controlled trial setting [38].

Recommendations for *N*-acetyl heparin (NAH) Pharmacological DVT prophylaxis should be applied in all critically ill COVID-19 known or rule-out cases barring contraindications (active bleeding, PLT < 25).

**Patients with no known thrombus and a D-dimer < 1000:**

- Consider initiating standard prophylaxis with enoxaparin 0.5 mg/kg SQ daily or heparin 5000 units SQ Q8H (or weight-based of 70 units/kg AdjBW if obese) in patients with significant renal impairment. For enoxaparin, anti-Xa monitoring should be applied in cases of extremes of weight, mild/moderate renal dysfunction, or elderly (> 65yo).
- Monitor coagulation parameters, including D-dimer Q48H.

**Patients with no known thrombus and a D-dimer ≥ 1000:**

- Consider initiating high-dose prophylaxis with enoxaparin 0.5 mg/kg SQ Q12H or heparin 7500 units SQ Q8H (or weight-based of 70 units/kg adjusted BW if obese) in patients with significant renal impairment.
- Anti-Xa monitoring should be applied in all cases of high-dose enoxaparin prophylaxis in this population
- Monitor coagulation parameters, including D-dimer Q48H.
- Consider reducing to standard prophylaxis if D-dimer resolves

**Patients with known or suspected thrombus**, or acute change in oxygenation, ventilation, hemodynamic status, or end-organ function:

- Consider initiating full-dose anticoagulation of enoxaparin 1 mg/kg SQ Q12H or heparin drip (may also consider heparin SQ at therapeutic dosing: 333 units/kg SQ × 1, followed by 250 units/kg SQ Q12h, with Xa levels 6 h after a dose at SS—pharmacists have access to this protocol).
- Anti-Xa monitoring should be applied in all cases of treatment-dose enoxaparin in this population
- Alternatively, may consider alteplase 25 mg IV over 2 h, followed by 1 mg/h for 25 h in known pulmonary thrombosis or acute decompensation.

## Conclusions

Due to its rapid emergence and spread, evidence surrounding COVID-19-associated bleeding disorders is still lacking. Of the proposed hypercoagulability markers, D-dimer testing appears to be a key laboratory test to monitor coagulability and predict patient prognosis. Monitoring parameters such as fibrinogen, PT and PTT are not reliable in this population. Despite the debate on the most appropriate approach in terms of thromboprophylaxis in COVID-19 patients, there is not enough evidence to support the use of higher than standard prophylactic doses and there are still concerns that patients treated with therapeutic doses may still experience thrombotic events. Indication of potential in situ pulmonary thrombi brings uncertainty to whether anticoagulant prophylaxis is even appropriate in the first place and increased doses may result in unnecessarily greater adverse effects for patients. More robust research is required to support the use of other treatment or preventative modalities such as plasma exchange.

## Data Availability

Data sharing is not applicable to this article, as no datasets were generated or analyzed during the current study.
